# Current concepts and future perspective of muscle function tests to inform return to sport decision‐making after ACLR: A narrative review

**DOI:** 10.1002/jeo2.70643

**Published:** 2026-01-21

**Authors:** Rebecca Hamrin Senorski, Ramana Piussi, Johan Högberg, Axel Sundberg, Jakob Lindskog, Robert Prill, Eric Hamrin Senorski

**Affiliations:** ^1^ Unit of Physiotherapy, Department of Health and Rehabilitation, Institute of Neuroscience and Physiology, Sahlgrenska Academy University of Gothenburg Gothenburg Sweden; ^2^ Sahlgrenska Sports Medicine Center Gothenburg Sweden; ^3^ Center of Orthopaedics and Traumatology University Hospital, Brandenburg/Havel, Brandenburg Medical School Theodor Fontane Brandenburg an der Havel Germany; ^4^ Faculty of Health Sciences Brandenburg Brandenburg Medical School Theodor Fontane Brandenburg an der Havel Germany; ^5^ Swedish Olympic Committee Stockholm Sweden

**Keywords:** ACL reconstruction, hop performance, muscle function test, muscle strength, return to sport

## Abstract

An injury to the anterior cruciate ligament (ACL) can be sustained in sports participation during situations such as offensive cutting, landing from a jump, or defensive pressing. Despite treatment with surgical reconstruction and/or rehabilitation, only 55%–84% of patients return to sport (RTS) after treatment, and second ACL injury rates remain as high as 25% in young athletes. To reduce the risk of second ACL injury, clinicians commonly use muscle strength tests, hop tests, and patient‐reported outcomes (PROs) to evaluate readiness for RTS. These tools have become central in clinical decision‐making, yet significant challenges remain. Current tests typically rely on limb symmetry index (LSI) thresholds, with ≥90% LSI commonly recommended as a clearance criterion for RTS. However, these cut‐offs have shown poor predictive value for second ACL injury risk and may be misleading. The LSI also fails to account for pre‐injury strength, bilateral deficits, or compensatory movement strategies. Hop tests provide additional insights into explosiveness and load absorption but emphasise distance or repetitions rather than movement quality, missing key biomechanical risk factors. Similarly, while PROs aim to capture the psychological dimension of recovery, they cannot be used in isolation to determine readiness for RTS. Collectively, these evaluation tools provide only snapshots of recovery and do not adequately reflect the chaotic, multidimensional demands of sport. The research gap lies not in the absence of tests, but in their limited transferability, standardisation, and contextual relevance. Current tests should not be viewed as a simple pass‐or‐fail clearance point, but as guidance for informed decision‐making in rehabilitation. Continuous evaluation across physical and psychological domains can help clinicians tailor progression, motivate patients, and support safer transitions toward sport. This narrative review explores the current state, limitations, and future directions of RTS testing in order the bridge the gap between clinical measures and the chaotic, high‐demand realities of sport.

**Level of Evidence:**

Level III.

**Abbreviations**
ACL: anterior cruciate ligament
ACL‐QoL: quality of life outcome measure for ACL deficiency
ACL‐RSI: anterior cruciate ligament‐return to sports after injury
EQ‐5D: EuroQol 5 Dimensions
ESSKA: European Society of Sports Traumatology, Knee Surgery and Arthroscopy
H/Q strength ratio: hamstings to quadriceps strength ratio
IKDC‐SKF: International Knee Documentation Committee Subjective Knee Form
KOOS: Knee injury and Osteoarthritis Outcome Score
K‐SES: Knee‐Self Efficacy Scale
LSI: Limb Symmetry Index
MARS: The Marx Activity Rating Scale
MCID: Minimal Clinical Important Difference
MIC: Minimal Important Change
PASS: Patient Accepted Symptom State
PROs: Patient Reported Outcomes
RFD: Rate of Force Development
RTS: Return To Sport
Tegner: Tegner activity scale
TSK: Tampa Scale of Kinesiophobia

## INTRODUCTION

An anterior cruciate ligament (ACL) injury is a serious knee injury frequently sustained in sports, especially among female athletes [12]. The ACL injury typically occurs as a non‐contact injury within 40 milliseconds(ms) from initial ground contact [50, 55] and often with the knee in slight flexion (20°–40°) alongside dynamic valgus [15]. The situational patterns for ACL injuries include offensive change of direction, defensive pressing, landing from a jump or regaining balance after a kick [15, 103]. At the estimated ACL injury occurrence during these patterns, athletes often display ipsilateral trunk tilt and contralateral rotation, hip abduction, dynamic knee valgus, and an externally rotated foot [15]. The kinematics of the ACL injury results in a mechanical load that exceeds the ACL′s capacity [65], which ultimately results in ACL rupture. Nonetheless, a large proportion of ACL injuries can be avoided through the general implementation of knee injury reduction programmes, such as the ESSKA‐ESMA ACL Prevention for All Program [82].

Treatment options for ACL injuries are rehabilitation alone or surgical reconstruction, with subsequent rehabilitation [8, 101]. The surgical reconstruction is performed with an autograft, most commonly with the hamstrings, patellar, or quadriceps tendon, or with an allograft. An additional lateral extraarticular tenodesis can also be added for further rotatory stabilisation [44]. The rehabilitation as part of the ACL treatment aims to restore muscle strength, hop performance, movement quality, perceived knee stability and knee‐related self‐efficacy, all of which are considered fundamental before RTS [69].

The rates of RTS ranges from 55% to 84% after ACL reconstruction [5, 45, 96], whereas only 14% of patients treated with rehabilitation alone return to competitive sports [46]. Although some patients are able to return to competition, others RTS with lower performance capacity [95], and a minority of patients who feel insecure may want a knee brace for extra external stability during sport participation [27]. The considered good RTS rates also have downsides, such as decreased level of activity [19], and incidences rates up to 47.5% of second ACL injuries in paediatric and adolescents [93]. In an attempt to minimise the risk for second ACL injury, clinicians use muscle strength and hop performance tests, together referred to as muscle function tests, to ascertain knee function recovery prior to RTS [34]. In addition to physical status, a patient's psychological status has been highlighted as an important factor prior to RTS [3]. As a result, psychological profiling and physical function are often recommended to be integrated into the battery of tests to ensure that clinicians assess a more complete picture of recovery to inform risk assessments and to support decision‐making for RTS under rehabilitation [21, 72]. To pass a battery of physical tests may decrease the risk for second ACL injury [34], however, results are inconclusive [105].

There is a plethora of tests currently used for testing, but what to assess, both physically and psychologically, and how results should be interpreted to best support each patient's strive to RTS with minimal risk for second ACL injury remains unclear.

## SCOPE

This narrative review offers an overview of current concepts, interpretations as well as future aspects of physical and psychological testing to inform RTS decision‐making for patients after ACL injury with or without subsequent reconstruction.

## STRUCTURED TESTING TO INFORM RTS DECISION‐MAKING

### Muscle strength

#### Current concepts

**Figure 1:**
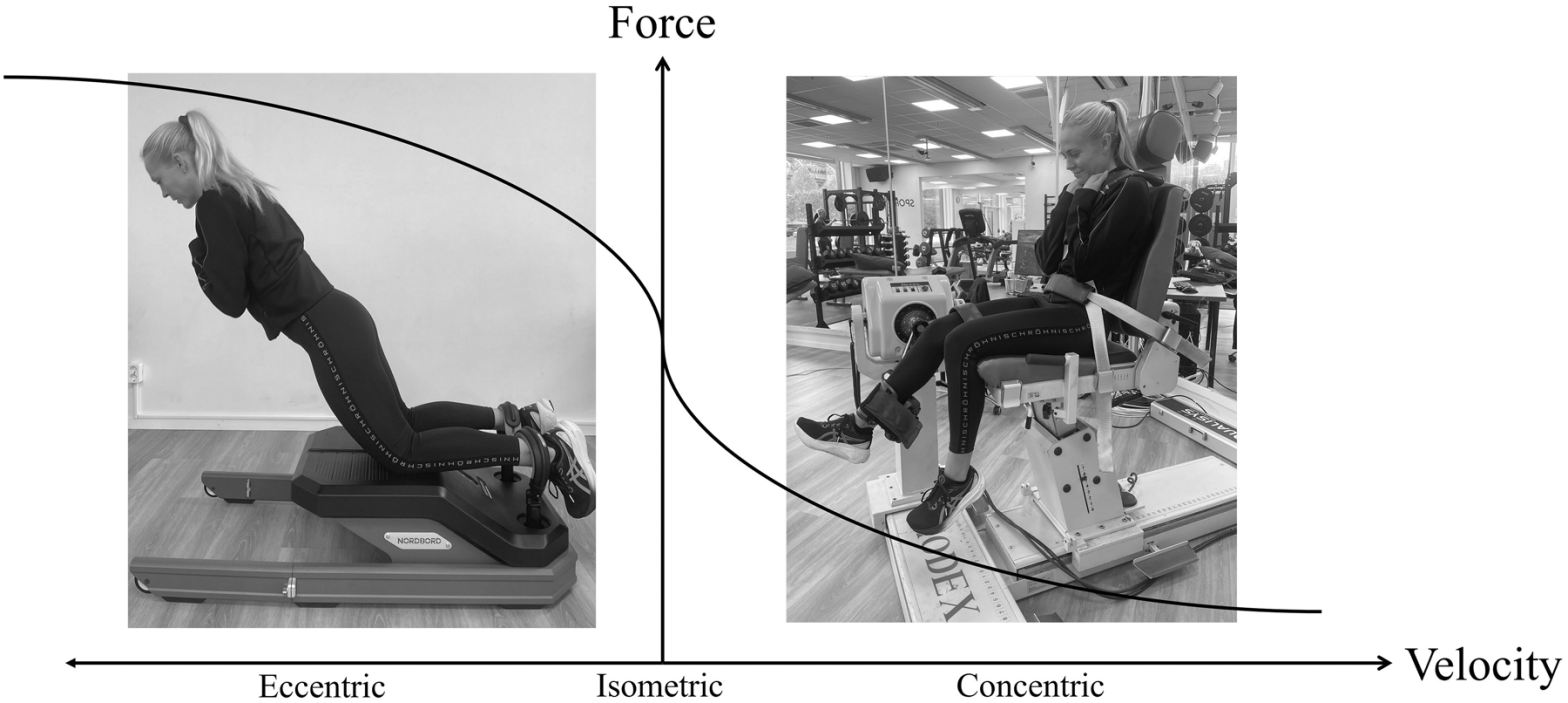
Force–velocity curve with eccentric, isometric and concentric contractions.

Knee flexion and extension muscle strength are of specific interest, as they provide stability to the knee joint by co‐contraction [9], and play an important role in facilitate deceleration effectively at landing and prior to cutting manoeuvres in sports [40]. Muscle strength is most frequently tested with isometric or isokinetic dynamometers, which are closely correlated for testing knee extension and flexion strength [39]. Isokinetic strength testing has become the preferred method for muscle strength evaluation, particularly in sports medicine, due to its standardised yet dynamic assessment and the ability to measure torque at multiple joint angles [99]. Force output from isometric contractions typically exceeds concentric contractions, but is generally lower than the force output during eccentric contractions (Figure 1).

Isokinetic muscle strength testing is typically performed concentric at angular velocities 60°–180° per second, where slower angular velocities produces greater peak torque than faster angular velocities [99]. For optimal concentric peak torque assessment, five repetitions of maximal effort are recommended at lower angular velocities [99]. Higher angular velocity than 60° per second may be considered after early ACL reconstruction or in the presence of patellofemoral knee pain, to reduce patellofemoral joint compression and anterior tibial translation induced by the quadriceps [108].

Isometric tests are user‐friendly and easily applicable in clinical practice [100]. Measures of isometric strength are recommended with a fixed dynamometry rather than with a hand‐held dynamometry. Measurement should be made at the knee flexion angle of the most beneficial length‐tension relationship for the respective muscle tested, where patients maintain force output for 5 s [68]. Commonly, the quadriceps muscle group produces maximum force at 60°–70° of knee flexion [86], whereas the hamstring muscle group at 20°–30° of knee flexion [47]. In cases where neither isokinetic nor isometric dynamometry is accessible, another option is to perform isotonic assessment in a weight machine using one repetition maximum, which displays high‐quality of evidence for sufficient criterion validity both for knee extension and flexion strength [100]. However, clinicians should be aware of that one repetition maximum leg extension test in an isotonic weight machine may overestimate quadriceps strength symmetry compared to isokinetic testing [90].

#### Interpretation of results

Muscle strength recovery is typically assessed as the strength relative to body weight, and as a symmetry between the injured and uninjured limb, that is, LSI calculated as $\frac{\mathrm{injured}\;\mathrm{limb}}{\mathrm{uninjured}\;\mathrm{limb}}\times 100\;=\mathrm{LSI}\%$. A common LSI threshold used to inform RTS decision‐making is ≥90% LSI based on expert opinions [54] and consensus statements [7, 62]. Although greater quadriceps strength is deemed important for running and an effective movement pattern in sports, a greater relative quadriceps strength can also impose a greater risk for second ACL injury in the absence of symmetry (≥90% LSI) [36, 87]. To achieve ≥90% LSI for quadriceps muscle strength, as well as in a battery of tests that include muscle strength and hop performance, has previously been reported to reduce second ACL injury risk by 84% [34]. However, a cohort study on young athletes reported that LSI cut‐offs, regardless of pre‐defined cut‐offs or in deviation from 100% symmetry, had poor predictive ability for a second ACL injury [89]. The LSI does not account for muscle strength decreases or increases of the uninjured limb, nor for preinjury strength of the reconstructed limb, which may bias the interpretation [107]. In addition, patients treated non‐operatively recover LSI faster compared to patients treated with ACL reconstruction [85], which may be due to the absence of donor‐site morbidity for patients treated non‐operatively. Patients treated with hamstring tendon autograft recover muscle strength LSI in knee extension and flexion faster than bone‐patellar tendon‐bone autograft, which may risk a premature RTS without adequate graft maturation [66]. Thus, LSI may be used as part of the interpretation of strength recovery through rehabilitation and as guidance towards RTS.

Besides LSI, the ratio between knee flexion (hamstring) to knee extension (quadriceps) strength (H/Q strength ratio) has been of interest, as the force relationship between these muscle groups could affect the tensile forces on the ACL. The hamstrings muscle group are considered a synergist to the ACL through a posterior shear force of the tibia on the femur (above 20°–30° of knee flexion), while the quadriceps induce an anterior tibial translation. A lower concentric H/Q strength ratio has been deemed a significantly greater risk factor for second ACL injury [58], and that this risk can decrease by 3% for every 1% increment of concentric H/Q strength ratio [42]. Importantly, similar to peak torque, the H/Q strength ratio differs between angular velocities, where lower velocities yield a lower H/Q strength ratio and greater velocities yield a greater H/Q strength ratio [10]. However, the H/Q strength ratio has limited predictive value as an independent risk factor for second ACL injuries [48]. The H/Q strength ratio should be similar to LSI peak torque measures, be used as a component in a comprehensive battery of tests in progression towards RTS and not as a determinant for RTS.

**Figure 2:**
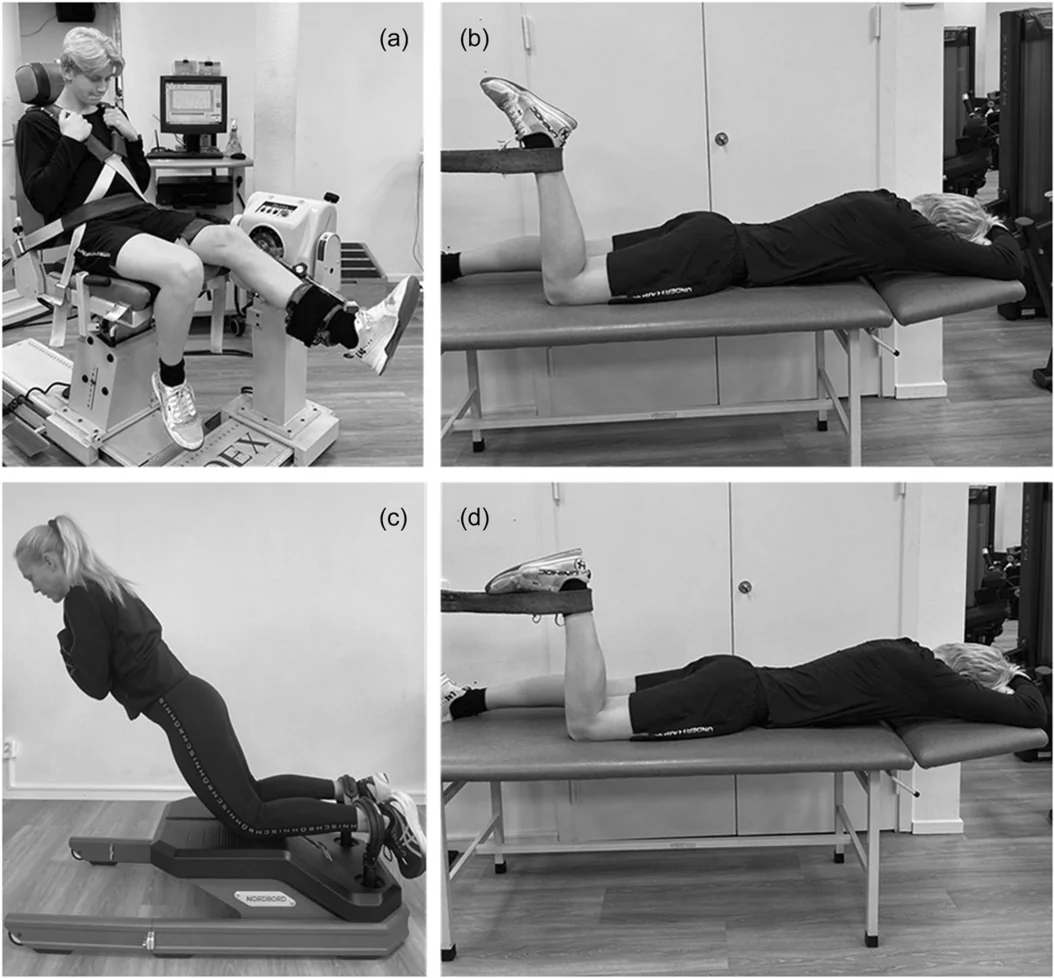
Illustration of different knee flexion testing positions. (a) Isokinetic knee flexion can be performed at different velocities in a seated position. (b) Isometric knee flexor strength test with hip in extension, knee flexion at 90° and ankle in plantar flexion. (c) Eccentric Nordic hamstrings strength test with hip in extension. (d) Isometric knee flexor strength test with hip in extension, knee flexion at 90° and ankle in dorsiflexion.

Since the quadriceps and hamstrings muscle groups are bi‐articular muscle groups, they activate differently depending on the hip position. Specifically, knee flexion tests (Figure 2) with the hip extended in prone/supine or kneeling positions may be more sensitive to identify residual knee flexor strength weakness not captured by traditional seated isokinetic test [41, 75]. The hamstring muscles contribute greatly to knee flexor strength output with the hip in flexed (seated) position, whereas the semitendinosus, biceps femoris short head, and gracilis have a greater impact on muscle force production relative to semimembranosus and biceps femoris long head with the hip in extension [51, 70]. Thus, it is important to be aware of the influence of different hip and knee positions when testing knee flexor muscle strength, and that one single position may not be sufficient to accurately reflect knee flexor strength and function.

### Hop performance

#### Current concepts

Hop tests complement strength assessments to reflect dynamic strength capacities, such as explosiveness, ground reaction force absorption, plyometric ability, and movement quality [13]. Several hop tests have been evaluated for their ability to discriminate between patients treated with ACL reconstructed and healthy controls, but methods and standardisation vary across the literature. Many hop tests have been described in the literature such as countermovement hop, hop for distance, side hop, crossover hop, triple hop for distance, 4 single‐legged hop, 5‐jump, drop vertical jump, tuck jump and 6‐metre timed hop [14, 25, 28, 35, 38, 60, 104, 106]. Countermovement hop, hop for distance, and side hop has been identified as the most effective tests to discriminate between the ACL‐injured and non‐injured limb in patients with an ACL injury [38].

Hop test evaluations can provide valuable insights into knee function but have inherent limitations when only assessed objectively with absolute or relative values and may thus risk to miss alterations in movement patterns. To capture alterations in movement patterns, the 'quality first' assessment can be used. The quality first assesses shock absorption, trunk, hip, knee and ankle joint positions from initial contact until the body's lowest center of gravity is reached, scored between 0 and 3 where a higher score indicate a greater movement quality [104].

#### Interpretation of results

Fifty four percent of patients who have suffered ACL injury exhibit asymmetries across multiple hop tests, such as single forward hop, triple forward hop, six metre timed hop test and triple cross over, side hop and vertical hop compared to healthy controls [29, 38]. Asymmetries in hop height, concentric impulse, and concentric/eccentric impulse ratio can also differentiate ACL reconstructed patients from healthy controls in countermovement hop and drop jump tests [59], whereas lower hop heights and higher dynamic knee valgus at landing can increase the odds of a second ACL injury [73]. Furthermore, case‐control data report that patients who sustained an ACL injury have deficits in both the involved and uninvolved limb compared with age‐ and sex‐matched norms [32].

Higher LSI in single‐ and repeated‐forward hop have been associated with greater odds for RTS and self‐reported symptoms and function after ACL injury [106]. Patients who achieve shorter hop distances may not necessarily have reduced strength or functional capacity but may instead lack confidence or trust in their knee [1]. Worse landing biomechanics, for example, greater knee extension and peak vertical ground reaction force, in jump‐landing tasks were associated with lower knee‐related self‐efficacy [49]. Patients who have undergone ACL reconstruction demonstrate reduced power production in the take‐off phase in hop tasks, as well as impaired control in deceleration upon landing, which may predispose patients to second ACL injury upon RTS [16, 24, 73]. On the other hand, patients who sustained a second ACL injury achieved longer hop distances (hop for distance and 5‐jump tests) and performed more hops in the side hop test on both limbs prior to their second injury, compared to patients who did not sustain a second ACL injury [25], which may reflects greater confidence and risk appraisal in addition to greater hop performance. Despite symmetrical hop test results, alternate movement patterns may be present, which could limit the use of LSI as a single variable for interpretation of results. This highlights the need to consider both quantitative and qualitative variables when evaluating hop performance in order to inform RTS decision‐making.

**Figure 3:**
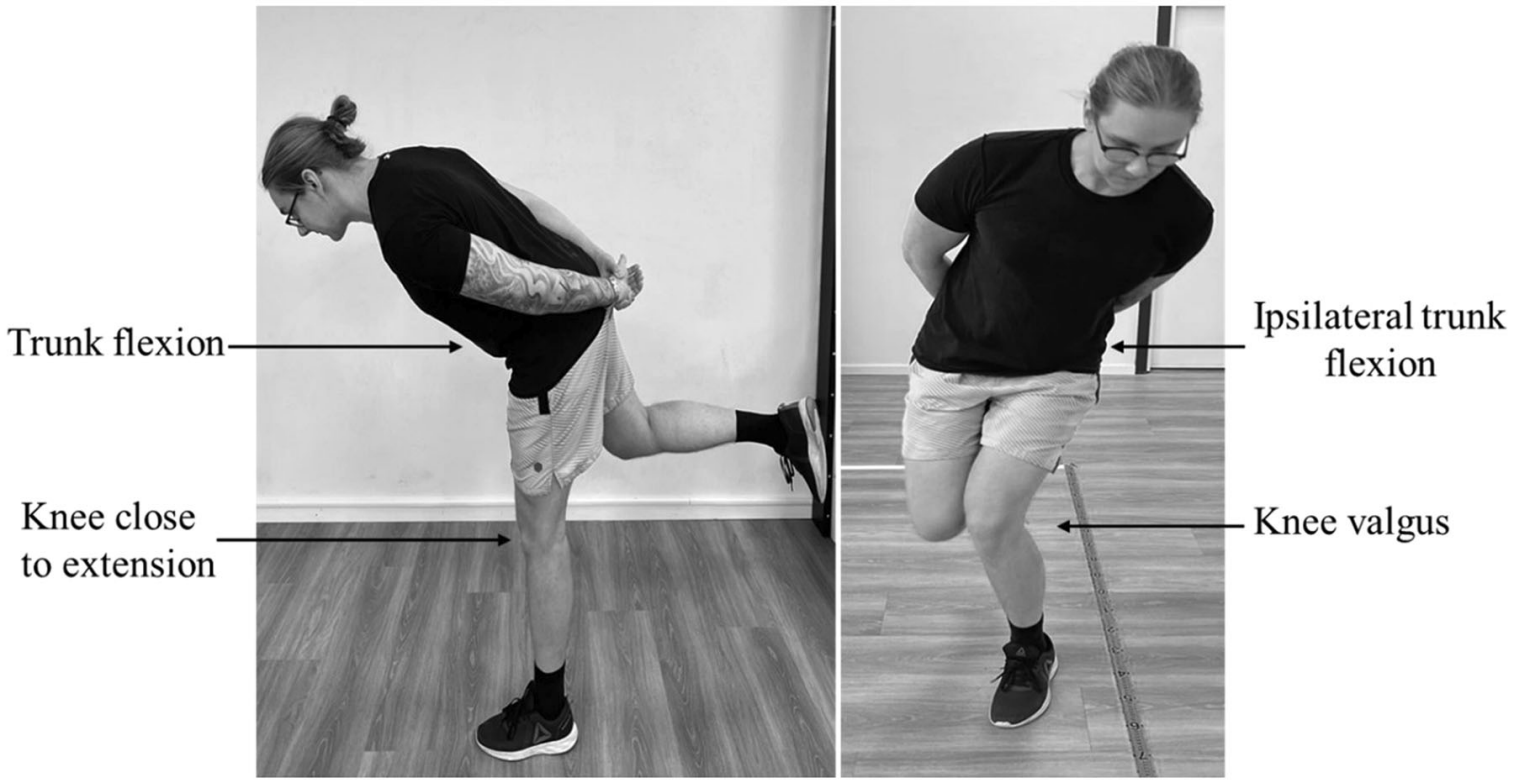
Compensatory movement patterns that can be observed in the hop tests which include great trunk flexion and knee close to extension as well as ipsilateral trunk flexion and knee valgus.

The conventional hop for distance test typically does not assess the movement quality of the landing phase, as it focuses only on hop distance. A greater quadriceps strength supports safer single‐leg landings by enabling greater knee flexion angle. Through the ability to absorb ground reaction forces through knee flexion while maintaining pelvic and upper body stability, is considered crucial for strain reduction on the ACL [16]. Patients who sustained an ACL injury can have a stiff landing and are thus more prone to compensatory movement patterns, such as increased hip flexion, knee valgus, forward trunk lean, and ipsilateral trunk flexion (Figure 3) [43, 52, 53]. Compensatory movement patterns underline the need to evaluate movement quality, force impulse, and deceleration qualities in combination with LSI and absolute values.

### Patient‐reported outcomes

#### Current concepts

Patient‐reported outcomes assess patients' perceptions of their physical and psychological status during and after ACL rehabilitation [2, 6]. Assessment of PROs is critical as patients can report an impaired quality of life despite recovery of physical function [61]. There are some overlaps in what the different PROs measure. To choose the right PRO for the target outcome measure can be a challenge, as there are many PROs that aim to evaluate the same construct. For instance, activity level can be measured with Tegner activity scale (Tegner), Lysholm score or Marx activity rating scale (MARS), while knee symptoms can be measured with the Knee Injury and Osteoarthritis Outcome Score (KOOS), International Knee Documentation Committee (IKDC) or ACL‐quality of life (ACL‐QoL). Confidence towards RTS is often measured with the ACL‐RSI which was rated in 2016 as the highest quality PRO in the evaluation of patients who suffered an ACL injury [26].

Despite consensus criteria that suggest that PROs should be included in batteries of tests to inform RTS decision‐making in ACL rehabilitation, few studies have attempted the integration of PROs into batteries of tests [78, 83]. Fear of re‐injury, lack of confidence in the knee, symptoms of depression, and loss of interest are psychological key factors that contribute to patients' inability to choose not to RTS [74, 92]. Clinicians play an important role in the evaluation and support of the patients' psychological well‐being to improve patients' psychological recovery after ACL injury or reconstruction prior to progression to sports.

#### Interpretation of results

Patients who successfully RTS at 12 months report higher ACL‐RSI scores at 6 and 12 months than those who did not [94]. Higher ACL‐RSI scores have also been associated with a 3% greater likelihood of RTS at 1 year [22, 91] and to return to pre‐injury activity level within 2 years from ACL injury [23]. A threshold of ≥65 has been reported as optimal ACL‐RSI score for RTS at 2‐year follow‐up [84]. One study [67] reported that an ACL‐RSI score ≤ 76.7 had 90% sensitivity to identify younger patients who sustained a second ACL injury, while another study reported that patients who sustained a second ACL injury had greater ACL‐RSI scores, implying greater confidence and risk appraisal towards RTS, compared to patients who did not [79]. While PROs such as ACL‐RSI can support RTS decision‐making after ACL injury, current evidence supports their use in combination with objective functional tests rather than as standalone tools.

Patients who achieve symmetrical muscle strength and hop performance ( ≥ 90% LSI) have greater knee‐related self‐efficacy and quality of life at both early (10 weeks) and later (12 months) follow‐ups after ACL reconstruction. While high knee‐related self‐efficacy and low fear of re‐injury may facilitate rehabilitation outcomes [57, 97], it could plausibly predispose patients to risk behaviours such as a premature RTS. Clinicians should be aware of risk behaviours in patients with high self‐efficacy and should thus assist these patients in understanding their limitations in order to avoid premature RTS.

Physical objective och psychological subjective recovery do not need to correlate and therefore clinicians could use patient accepted symptom state (PASS), minimal important change (MIC) or minimal clinical important difference (MCID) to interpret changed PRO scores [63, 71, 81]. By incorporating PASS, MIC or MCID alongside objective tests of physical function such as hop tests strength tests, and movement quality clinicians can determine whether improvements in psychological status are meaningful from the patient′s perspective and not just if patients achieve objective thresholds [76]. Clinicians should therefore integrate PASS, MIC or MCID for PROs with objective measurements to further assist in progression towards RTS.

## WHEN TO TEST

Current measurements to guide progression towards RTS are simplified but widely used approaches to estimate whether a patient can be ready for RTS with the lowest possible risk for second ACL injury, but the tests provide only a snapshot of physical and psychological status, which may change over time. It is thus important to monitor patients continuously under the rehabilitation period and through the RTS continuum.

When muscle function tests and PROs are consistently used under the rehabilitation process, they can democratise rehabilitation where successful outcomes can be achieved at clinics with high and low volumes of patients [88]. Current batteries of tests should be regarded as one part of the rehabilitation process rather than as definitive criteria for RTS clearance. They are more appropriately used to determine whether patients are ready to progress towards more sport‐specific training, such as on‐field rehabilitation.

**Figure 4:**
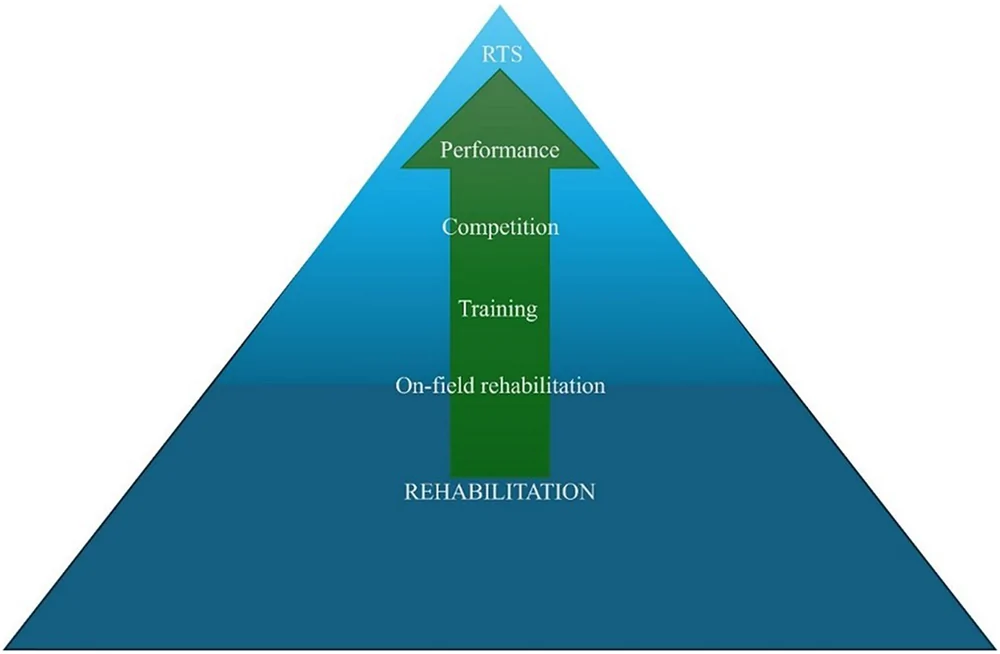
Illustration of on‐field rehabilitation phases towards the ultimate goal of return to sport. Rehabilitation is illustrated by the green arrow, and phases to go through are on‐field rehabilitation which include sport specific demands, training which include participation in sport specific training moments with the team, competition which include participation in competition on training, performance which include restricted participation in match play against opponents, which finally leads up to RTS which include full unrestricted participation in sport. RTS, return to sport.

On‐field rehabilitation [77] can strengthen the return‐to‐performance phase which refers to the gradual progression in restoration of sport‐specific skills and physical capacity before full competition to support a safer and more individualised transition back to sport, illustrated in Figure 4. This approach shifts perspective from RTS tests as a single clearance point to a continuum of testing under the rehabilitation process with the ultimate outcome of RTS. Tests should thus inform clinicians if patients are able to progress rather than give an absolute decision to resume competition.

Furthermore, timing of RTS has previously been reported as a factor that might influence the risk for second ACL injury. Beischer et al. [11] reported that patients who RTS < 9 months after ACL reconstruction had increased second ACL injury risk compared to patients who delayed RTS ≥ 9 months. A systematic review [80] also reported that patients who sustained a second ACL injury RTS earlier compared to patients who did not, however, no significant difference was found for elite athletes. Clinicians should prioritise individualised, criteria‐based testing, but consider time‐based milestones to ensure sufficient functional recovery prior to progression towards RTS.

## GAPS IN TESTING

Tests included in current battery of tests often lack transferability and validity in relation to the chaos of sports [105]. Missing components to current batteries of tests to reduce the gap between clinic and the dynamic nature of sports, components such as rate of force development (RFD), neurocognitive measures and on‐field tests are of interest [18, 37, 64].

### RFD

The RFD measures the ability to generate force rapidly and is typically measured under isometric conditions [13, 64]. Early RFD (0–100 ms) depends on the intrinsic ability of muscle fibres to activate quickly at ground contact, whereas peak torque is typically reached 150–300 ms after contact, for example during a change of direction [4]. Despite achieving acceptable LSI cut‐offs in the knee extorsion and flexion (≥90% LSI), patients with ACL reconstruction often demonstrate significantly lower RFD in the involved limb compared to the uninvolved limb and healthy controls [98]. Given that ACL ruptures usually occur within the first 50 ms of ground contact [56], RFD should be assessed and targeted throughout rehabilitation and RTS to ensure explosive muscle function is restored, which may be important for safer progression to sports.

### Neurocognitive measures

**Figure 5:**
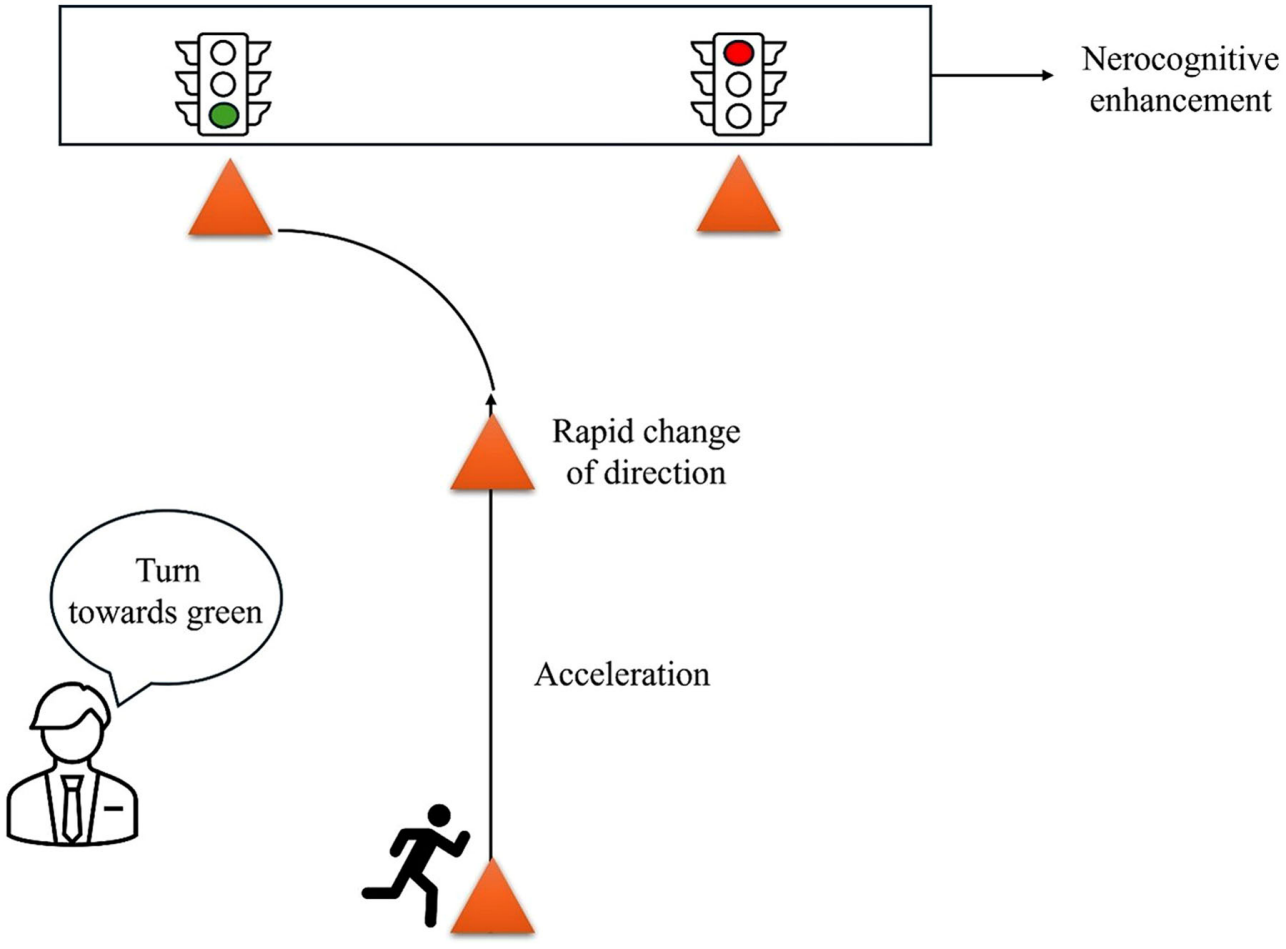
Illustration of an example of neurocognitive enhanced change of direction testing where patient starts accelerating towards a target and performs a rapid change of direction towards a sudden target. Traffic lights light up just prior to patients reach the cone where the change of direction occurs.

Injury to the ACL often occurs due to neurocognitive errors such as poor motor‐response, or attentional inhibition [31]. Current muscle function tests focus on an isolated task, for example, knee extension, whereas sport requires athletes to navigate complex environments and respond to external stimuli. Upon a neurocognitive demand in hops, patients significantly reduces their hop distance [33]. To incorporate cognitive challenges, or dual‐task conditions, helps bridge the gap between controlled clinical testing to the unpredictable, high‐demand nature of sport. An illustration of enhanced neurocognitive testing is presented in Figure 5.

### On‐field testing

A common movement pattern that leads to ACL injury involves rapid horizontal deceleration. Therefore, on‐field deceleration tests may be a key component prior to RTS, yet rarely included in discharge criteria [30]. On‐field assessments of change of direction drills (such as the 5‐0‐5 agility test or the t‐test) may reveal a movement pattern associated with an increased load on the ACL [17]. Modifications to the change of direction techniques may include encouraging earlier breaking during the penultimate foot contact, backward trunk inclination, neutral foot plant orientation and increased knee flexion. Given the variability in sport performance, on‐field tests should be designed to closely match the specific demands of the athlete's sport including RFD, change of direction, and neurocognitive demands.

## THE FUTURE OF TESTING

High quality research has implemented, structured and repeated muscle function tests to move away from the ‘guess’ to ‘measure’ in hope to better guide informed decision into progression towards RTS [102]. After RTS patients may need further testing in order to continue to perform on the field [20]. Tests of muscle function have an important role in ACL rehabilitation as these tests creates motivation in athletes and creates concreteness to the communication between health care professionals and athletes. Objective evaluation should be as comprehensive as needed for each individual patient, tailored to their specific need and context. Ideally, clinicians should be able to explore several dimensions of the patient's knee function after ACL injury or ACL reconstruction which include strength (LSI, relative strength, and RFD), biomechanics (including movement quality), neurocognitive functions and psychological dimensions. Table 1 presents a summary for recommendation, suggested tests and interpretation option for each test.

**Table 1:** Summary for recommendation, suggested tests and interpretation for each test.

Assessment type	Suggested tests	Variables	Interpretation
Muscle strength	Isokinetic (60°–180°/s), isometric, isotonic 1RM (if no dynamometer)	Use LSI, relative strength, H/Q strength ratio, endurance, RFD Symmetry alone insufficient	≥90% LSI for RTS (limited predictive value) Relative strength ~3.0–3.5Nm/kg knee extension and ~1.20–1.50 Nm/kg knee flexion H/Q strength ratio ~60% (angular velocities ≤60° per second)
	Test in multiple hip/knee positions		
Movement quality/biomechanics	Two‐ and three‐dimensional analysis of cutting, jumping, and other sport‐specific movements.	Assess trunk, hip, knee, and ankle angles for compensatory movement strategies	Look for stiff landing with knee close to extension, trunk lean in flexion or lateral flexion, and knee valgus Altered movement strategies, e.g., excessive trunk flexion or knee valgus can increase the risk for second ACL injury
Neurocognitive measures	Tests that simulate sport‐specific cognitive load with imposed external focus (e.g., dual‐tasking, reactive drills, colour response).	Performance under cognitive load Mimic sport demands and assess movement patterns under sports‐realistic stressors.	Hop symmetry (≥90% LSI) under sport specific cognitive load Delayed response time in, e.g., colour response tasks compared to uninjured side Altered movement strategies, e.g., excessive trunk flexion or knee valgus
Psychological dimensions	Assess knee confidence, stress response, life stressors, recovery from stressful events.	PROs such as PROs: ACL‐RSI, K‐SES, TSK, KOOS‐QoL, IKDC‐SKF	Both high and low psychological scores can lead to second ACL injury risk Track MIC, MCID, PASS for meaningful change Integrate with physical tests
On‐field test	Deceleration and change of directions drills, such as 5‐0‐5 agility test and T‐test.	Assess deceleration and change of direction performance and altered movement patterns in sport‐like situations	Altered movement patterns, e.g., excessive trunk flexion or knee valgus Final clearance for RTS requires integration between several assessment types and RTS should not decide using only one type.

Abbreviations: ACL, anterior cruciate ligament; ACL‐RSI, anterior cruciate ligament‐return to sports after injury; COD, change of direction; e.g., exempli gratia (for example); H/Q ratio, hamstrings to quadriceps ratio, IKDC‐SKF, International Knee Documentation Committee‐Subjective Knee Form; K‐SES, Knee‐related Self‐Efficacy; KOOS‐QoL, Knee injury and Osteoarthritis Outcome Score‐Quality of Life; LSI, Limb Symmetry Index; MCID, minimal clinical important difference; MIC, minimal important change; PASS, Patient Accepted Symptom State; RFD, rate of force development; RM, repetition maximum; RTS, return to sport.

Given the multifactorial nature of secondary ACL injury risk, no single test or battery of test, can fully account for all internal and external risk factors that constitute to an increased secondary ACL injury risk. It is a major challenge to accurately predict the occurrence of a second ACL injury using current test protocols alone. The tests should instead be used as continuous evaluation throughout rehabilitation to provide valuable guidance, support patient motivation, and help tailor progress towards RTS for each patient′s needs. Thorough successive exposure to sports with on field‐rehabilitation and testing, and by including all dimensions of evaluation (Table 1), the gap between clinic and sports participation may decrease. Furthermore, it is important to acknowledge time as a factor for RTS.

## SUMMARY

Return to sport after an ACL injury involves a multifaceted assessment of muscle function, biomechanics, psychological state, and emerging neurocognitive factors. Strength and hop performance tests are widely used, presented as absolute values, H/Q strength ratio but mostly as LSI. However, LSI has poor predictive ability for second ACL injury. Relative strength may provide additional insights, though there is no consensus on ideal cut‐off values, which also will vary depending on the demands of the sport as well as patient sex.

Hop tests, such as the countermovement hop and hop for distance, evaluate dynamic knee function but may mask underlying biomechanical deficits. To improve test accuracy, movement quality assessments like the “quality first” approach emphasise proper landing mechanics and force absorption, all essential for sports participation.

Confidence and risk appraisal towards RTS, fear of re‐injury, and knee‐related self‐efficacy are key factors that influence RTS outcomes. PROs such as the ACL‐RSI are highly regarded for assessment of mental status. However, inconsistencies in PROs psychometric properties make it difficult to recommend the single best tool for evaluation.

Despite improvements in tests to inform RTS decision‐making, traditional measures do not fully bridge the gap between clinic and the complex and unpredictable demands of sports. Emerging assessments such as RFD, neurocognitive tests, and on‐field evaluations offer new insights into ACL recovery.

## AUTHOR CONTRIBUTIONS

Authors Rebecca Hamrin Senorski, Robert Prill and Eric Hamrin Senorski were responsible for the design and outline of the review. First author Rebecca Hamrin Senorski was responsible for the first draft of the manuscript. Authors Johan Högberg and Ramana Piussi assisted greatly in the text functionality. Authors Axel Sundberg, Jakob Lindskog, Robert Prill and Eric Hamrin Senorski assisted in critically analysing the text for errors and helped finalise the manuscript. All authors contributed to the final draft of the review for submission.

## CONFLICT OF INTEREST STATEMENT

Author Eric Hamrin Senorski is the associate editor of Journal of Orthopeadic and Sports Physical Therapy. Authors Rebecca Hamrin Senorski, Ramana Piussi, Johan Högberg, Jakob Lindskog, Axel Sundberg or Robert Prill has no conflict of interest to disclose.

## ETHICS STATEMENT

None declared.

## Data Availability

None declared.
